# OmniCellAgent: Towards AI Co-Scientists for Scientific Discovery in Precision Medicine

**DOI:** 10.1101/2025.07.31.667797

**Published:** 2025-08-02

**Authors:** Di Huang, Hao Li, Wenyu Li, Heming Zhang, Patricia Dickson, Ming Zhan, J Philip Miller, Carlos Cruchaga, Michael Province, Yixin Chen, Philip Payne, Fuhai Li

**Affiliations:** 1 Institute for Informatics (I2),; 2 Computer Science & Engineering,; 3 Division of Statistical Genomics,; 4 NeuroGenomics and Informatics,; 5 Center for Translational Bioinformatics (CTBI),; 6 Department of Pediatrics, Washington University in St Louis, MO, USA.; 7 National Institute of Drug Abuse, NIH, MD, USA.

## Abstract

The convergence of large language models (LLMs), AIagents, and large-scale omic datasets—such as single-cell omics, marks the arrival of a critical inflection point in biomedical research, via autonomous data mining and novel hypothesis generation. However, there is no specifically designed agentic AI model that can systematically integrate large-scale single-cell (sc) RNAseq (covering diverse diseases and cell types), omic data analytic tools, accumulated biomedical knowledge, and literature search to facilitate autonomous scientific discovery in precision medicine. In this study, we develop a novel agentic AI, OmniCellAgent, to empower non-computational-expert users—such as patients and family members, clinicians, and wet-lab researchers—to conduct scRNA-seq data–driven biomedical research like experts, uncovering molecular disease mechanisms and identifying effective precision therapies. The code of omniCellAgent is publicly accessible at: https://fuhailiailab.github.io/.

## INTRODUCTION

The year 2025 is marked as the rise of AI agents - autonomous systems capable of perceiving, reasoning, and acting within an environment. Large language models (LLMs), trained on vast amounts of text data, demonstrate unprecedented capabilities in understanding context, generating human-like text, and performing complex reasoning tasks. Their potential impact on healthcare, and Medical Question and Answering (Q&A), medical scientific discovery, in particular, is pro-found (Wang et al. 2023). Biomedical discovery for disease treatment is a grand challenge at the intersection of artificial intelligence (AI) and healthcare because most people will die from complex diseases, which have unclear pathogenesis and no effective and curable treatment. Clinicians and researchers must sift through an ever-growing deluge of biomedical knowledge to find relevant and up-to-date answers for optimal patient care. For example, more than 1.5 million new articles in biomedicine and life sciences are published on PubMed each year (González-Márquez et al. 2024). This overload of information is particularly acute when determining treatment strategies for complex diseases, where one must integrate clinical guidelines, latest research findings, and patient-specific factors. An intelligent system capable of rapidly retrieving and reasoning over this vast knowledge base could dramatically improve decision making in precision medicine, ensuring that patients receive treatments aligned with the most recent evidence and their unique context. However, developing a reliable biomedical knowledge discovery system for treatment questions presents significant difficulties. State-of-the-art large language models (LLMs) (Jones 2025; Team et al. 2024) have shown a remarkable ability to answer general questions, but often hallucinate facts or rely on outdated knowledge, which is unacceptable in complex medical settings (Sohn et al. 2024).

In biomedical research, recent advances have explored agent-based systems for literature synthesis (Gao et al. 2024), hypothesis generation (Qi et al. 2024), and knowledge-driven discovery (Wang et al. 2023). Traditional approaches usually follow a single-agent pipeline – first retrieving documents from literature databases like PubMed, then extracting or generating an answer – which can limit adaptability (Jin et al. 2023). These systems may falter when queries require combining information or when the query is ambiguous and needs clarification. Recently, a more flexible and knowledge-based approach is proposed to address the breadth of treatment related questions (Gottweis et al. 2025). However, in these studies, large-scale single cell RNAseq datasets, biomedical knowledge and agent-specific knowledge that mimics the domain knowledge in individual laboratories were not well presented and integrated.

In other previous studies, we developed BioMedGraphica (BMG) (Zhang et al. 2024d) to integrate the rich human-understandable biomedical knowledge of multi-omic data and signaling pathways, which are helpful for LLMs to understand the relationships of given targets mined from large-scale scRNAseq datasets. Moreover, we develop omniCellTOSG (Zhang et al. 2025b), which collected the scRNAseq data of about a hundred of millions of single cells and meta-cells (a group of single cells). Based on these valuable data, in this study, we presen a novel agentic AI, OmniCellAgent, to empower non-computational-expert users—, uch as patients and family members, clinicians, and wet-lab researchers—to conduct scRNA-seq data–driven biomedical research like experts, uncovering molecular disease mechanisms and identifying effective precision therapies.

## RELATED WORKS

The task of answering biomedical questions, especially about disease treatments, has been tackled by various systems over the past decade. The advent of large pre-trained language models brought significant improvements. Models like BioBERT (Lee et al. 2020) and PubMedBERT (Gu et al. 2021) are domain-specialized transformers trained on biomedical corpora. However, even a fine-tuned biomedical LLM may fail to recall a specific up-to-date clinical trial result or guideline recommendation. To address this, researchers have turned to retrieval-augmented generation (RAG) (Lewis et al. 2020) techniques in the biomedical context (Liu et al. 2025). In the RAG setup, the model actively fetches relevant text snippets from external sources during answering. This has been shown to greatly reduce hallucinations and improve answer accuracy in medical Q&A (Li et al. 2025; Hu et al. 2024). To further enhance the capabilities of RAG systems, recent research has explored the integration of multi-agent reinforcement learning and agentic architectures (Singh et al. 2025). One such advancement is the Multi-Module joint Optimization Algorithm for RAG (MMOA-RAG) (Chen et al. 2025), which treats each component of the RAG pipeline—such as query rewriting, document retrieval, and answer generation as individual agents within a cooperative multi-agent reinforcement learning framework.

Recently, biomedical research is starting a paradigm shift with the integration of autonomous AI agents. They can participate in the scientific process by generating hypotheses, debating alternatives, and incorporating evidence, all while working alongside human experts. For example, Gottweis et al. (Gottweis et al. 2025) introduced “AI co-scientist”, a multi-agent system capable of proposing and refining novel research hypotheses in an autonomous loop. Similarly, scientists have envisioned biomedical AI agents that integrate with experimental platforms and use prior knowledge to drive discoveries without removing the human from the loop (Gao et al. 2024; Moritz et al. 2025). The introduction of dynamic tool use (Qin et al. 2023), where agents flexibly call external knowledge bases and text mining utilities, enables our system to handle complex queries that span the full spectrum of biomedical data. SpatialAgent (Wang et al. 2025), exemplifies this progression by offering a fully autonomous AI system tailored for spatial biology research. Using LLMs combined with tool execution and adaptive reasoning to navigate complex biological data, its capabilities span the entire research pipeline, from experimental design to multi-modal data analysis and hypothesis generation, demonstrating versatility across various tissues and species.

## METHODOLOGY

**Fig. 1.**
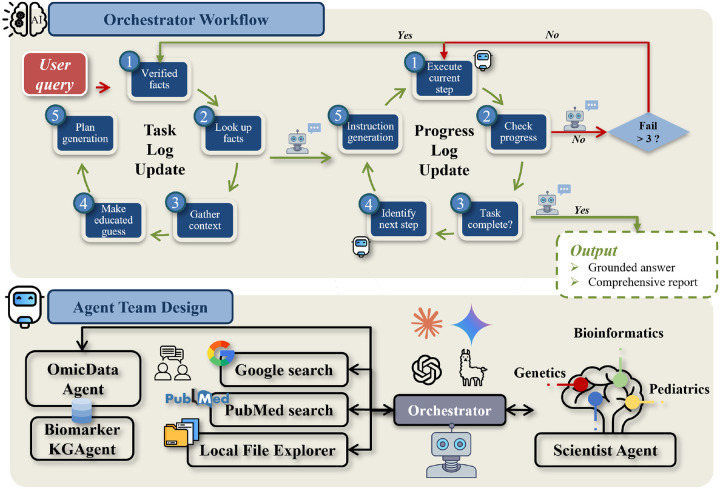
Overview of the Multi-agent Model Architecture.

We designed a multi-agent system, inspired by AutoGen (Wu et al. 2023), that coordinates specialized agents to solve complex problems. This architecture is governed by an Orchestrator agent that manages the entire workflow, from query to solution. The Orchestrator interprets the user’s query, devises a strategy, and directs sub-agents to execute the plan. It monitors progress and synthesizes intermediate results to generate the final output. Figure 1 illustrates this framework.

### Orchestrator

The Orchestrator operationalizes a user query by creating a dynamic Task Log. This log systematically deconstructs the query by: 1) retrieving known facts from its established knowledge; 2) identifying information gaps by comparing internal knowledge against the query’s demands; and 3) generating working assumptions to bridge these gaps. These assumptions function as preliminary hypotheses that guide the subsequent planning phase.

Based on this analysis, the Orchestrator generates a comprehensive Task Plan. This involves mapping the required actions to the specific capabilities of available agents, such as the Omic Data Agent, Scientist Expert Agent, Google Search Agent, and PubMed Paper Search Agent. The resulting plan outlines a detailed sequence of operations assigned to these agents, ensuring a coordinated and systematic approach. While these agents are capable of independently retrieving and analyzing information, the Orchestrator’s role is to ensure that their actions are aligned with the overall goal of answering the user’s query.

To manage the workflow, the Orchestrator maintains a Progress Log to record and monitor task execution and agent responses. For each step in the plan, the Orchestrator commands the designated agent and assesses its progress. Upon successful execution, it selects the next agent and instructs the next task outlined in the plan. If the current task fails, the Orchestrator commands the agent to retry up to a predefined threshold (e.g., three attempts). If the task fails persistently, the system initiates a reflection and self-refinement cycle: it analyzes the cause of failure, updates the Task Log with any newly learned information, and revises the Task Plan to.This adaptive execution loop continues until all steps in the plan are fulfilled. Finally, the Orchestrator synthesizes the findings gathered by all sub-agents to generate a comprehensive response that is structured to address the user’s original query.

The interplay between the Task Log, which defines the plan, and the Progress Log, which tracks its execution, forms the core operational loop. This allows the Orchestrator to systematically guide the multi-agent collaboration, dynamically adapting the plan based on new information to ensure all agent actions are coordinated to resolve the user’s initial query.

### Omic Data Agent

The OmicDataAgent is a specialized component designed to facilitate context-aware multi-omics analysis for biomedical research queries. Its primary functions include: (1) identifying and retrieving omic datasets relevant to a given biological or disease-related question from OmniCellTOSG (Zhang et al. 2025b), (2) performing robust statistical analyses and enrichment assessments, and (3) extracting key biological insights such as differentially expressed genes (DEGs) and significantly enriched signaling pathways and biological processes. OmniCellTOSG (Omni-Cell Text-Omic Signaling Graph) is a large-scale, graph-structured, AI-ready dataset that harmonizes single-cell transcriptomics data and biological knowledge graph, BioMedGraphica (Zhang et al. 2024d; Zhang et al. 2025a), across diverse disease contexts, tissue types, and cell populations. It integrates curated annotations, metadata, and multi-omics features from public repositories such as CellxGene (Program et al. 2025), BrainCellAtlas (Chen et al. 2024), and GEO (Edgar et al. 2002), enabling comprehensive representation of disease-relevant cellular states and signaling activities. The graph structure of OmniCellTOSG encodes both molecular attributes (e.g., gene expression profiles, pathway activities) and biological relationships (e.g., signaling pathways and protein-protein interactions), allowing intelligent agents to reason over complex omic landscapes.

When a user submits a natural language query, such as “What are the differentially expressed genes in microglial cells in Alzheimer’s Disease?”, the Omic Data Agent interprets the prompt to extract key biological entities, including the disease context, relevant cell type, and intended analysis objective. It then retrieves matched disease and control meta-cell datasets from OmniCellTOSG, a harmonized resource of single-cell omics profiles curated across diverse biological and pathological conditions. Using statistical models such as the Wilcoxon rank-sum test (executed with parallel processing), the agent identifies differentially expressed genes (DEGs) within the specified context.

These DEGs are subsequently analyzed through a functional enrichment workflow that programmatically interfaces with the Enrichr API. The agent submits gene lists and queries a wide array of curated databases, such as Gene Ontology (GO) (The Gene Ontology Consortium 2018), KEGG (Kanehisa and Goto 2000), Reactome, MSigDB, OMIM, and DisGeNET (Piñero et al. 2016) — to uncover enriched biological pathways and disease associations. The analysis pipeline includes automated gene list submission, structured retrieval and storage of enrichment results across multiple databases, generation of summary reports distinguishing pathway- and disease-based enrichments, and export of top-ranked terms prioritized by adjusted p-values, along with annotated gene counts and pathway memberships.

**Fig. 3.**
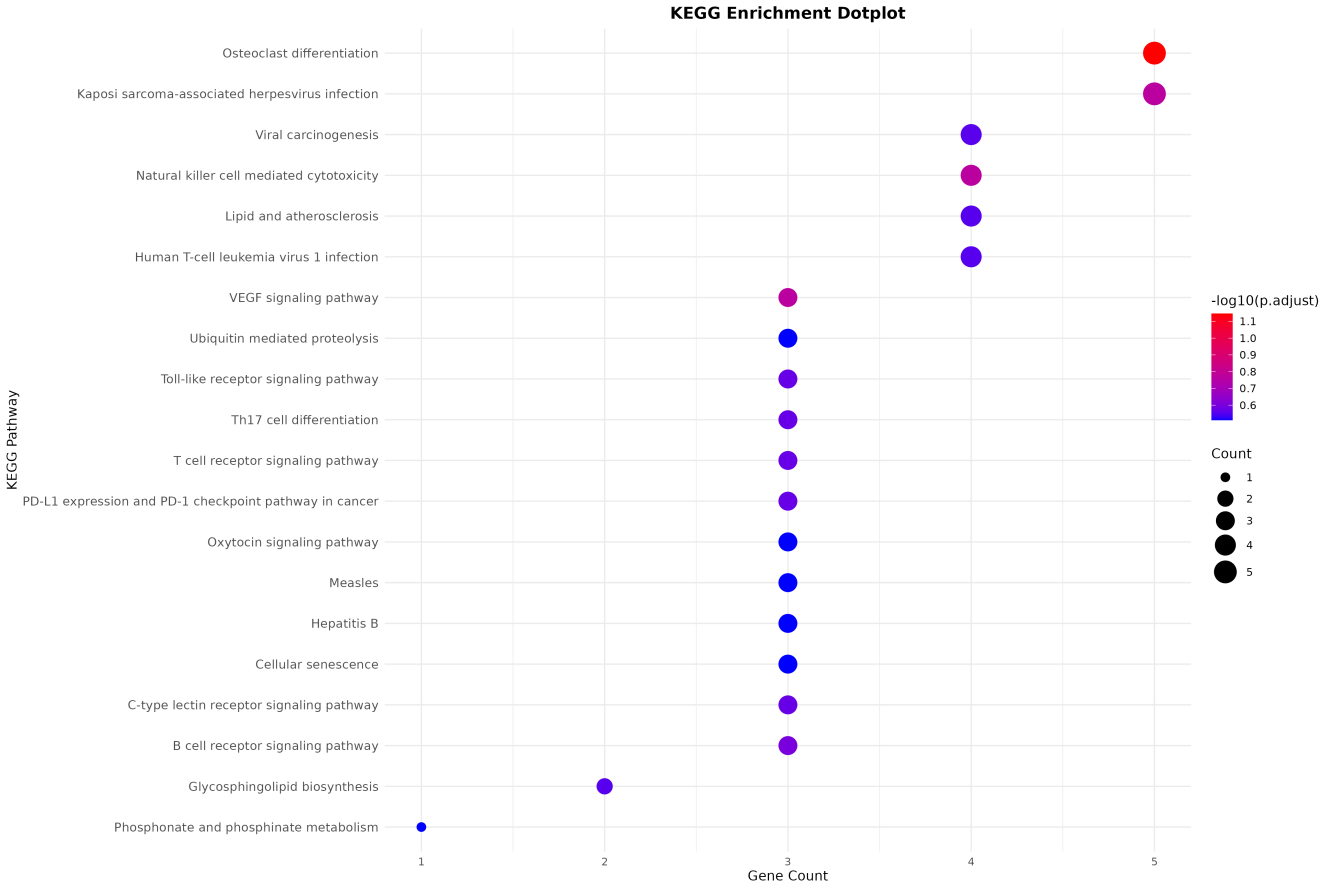
KEGG Enrichment Summary.

**Fig. 4.**
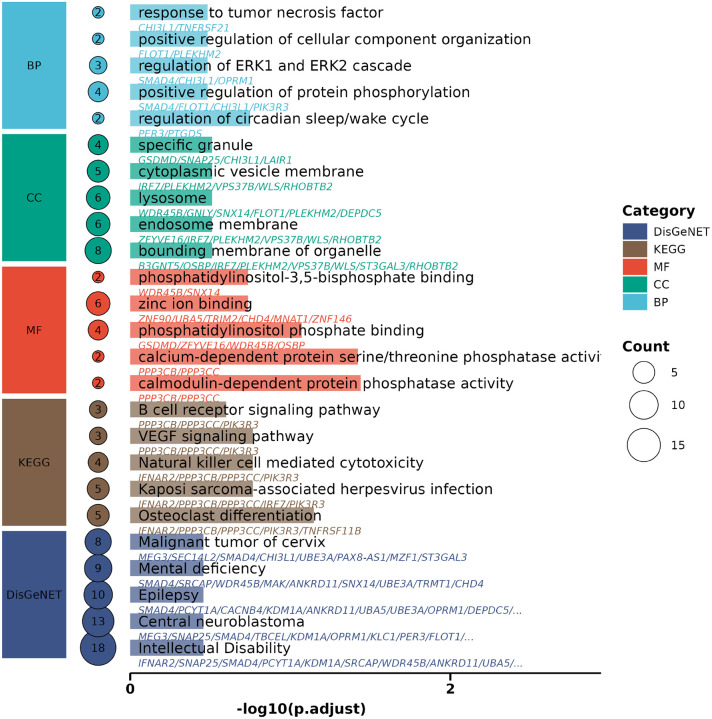
Vertical Pathway Bar Plot.

Following enrichment analysis, the resulting gene set enrichment tables across Gene Ontology (GO) categories—Biological Process (BP), Cellular Component (CC), Molecular Function (MF)—as well as KEGG pathways and disease associations (from DisGeNET) were consolidated into a unified data structure for visualization. Each result table contained pathway terms, associated gene counts, and adjusted p-values. In order to make comparative interpretation, the top five most significant terms (ranked by adjusted p-value and gene count) were selected within each ontology category. The output consists of three figures that collectively summarize functional and differential expression analyses:

1. KEGG Enrichment Summary: A dot plot highlighting the top enriched KEGG pathways, ranked by adjusted p-values. Dot size represents gene count per pathway, and color reflects statistical significance (−log_10_ (*p*-value)). Figure3 shows the example of KEGG enrichment summary.
2. Vertical Pathway Bar Plot: A structured vertical bar chart summarizing GO (BP, CC,MF), KEGG, and DisGeNET enrichments. Categories are color-coded and grouped, with pathway names and gene annotations displayed alongside each bar. The height of each bar corresponds to enrichment significance, and dot size indicates the number of associated genes. Figure 4 shows the example of vertical pathway bar plot.
3. Lollipop Plot of DEG: A lollipop chart representing the log_2_ fold change and associated p-value for selected differentially expressed genes. Each gene is shown as a point on a horizontal axis of log_2_ *FC*, with a stem encoding its statistical confidence. Figure 5 shows the example of lollipop plot of DEG.

### Scientist Expert Agent

The ScientistExpertAgent is designed to provide deep, specialized knowledge reflecting the cumulative work and historical perspective of a specific scientific expert. Its function is to serve as a focused repository of that individual’s contributions to their domain. The expert’s publications are routinely indexed and cached. This extensive corpus of scientific papers is processed and managed using the LightRAG framework (Guo et al. 2024). Within LightRAG, documents undergo several processing steps: they are parsed and segmented into text chunks, and dense vector embeddings are generated for these chunks to enable efficient semantic similarity searching across the large volume of text. LightRAG also constructs an internal knowledge graph from the ingested papers. This graph captures entities and relationships discussed within the expert’s work, offering a structured, multi-dimensional representation of their knowledge landscape beyond simple text retrieval.

**Fig. 6.**
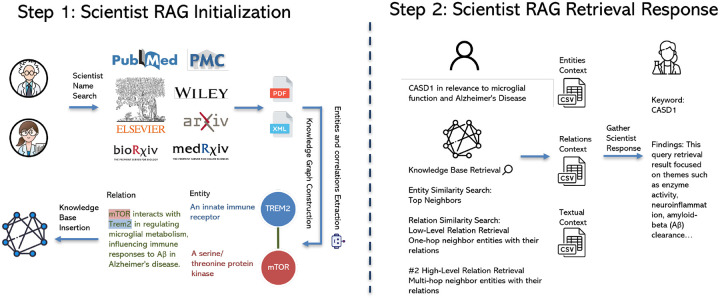
Overview of the Scientist Expert Agent Workflow.

Upong receving a query, the Scientist Expert Agent retrieves relevant documents from its indexed corpus and generates a response based on the expert’s accumulated knowledge. This response is tailored to reflect the expert’s unique insights and perspectives, providing users with a specialized understanding of the topic at hand. Figure 6 illustrates the workflow of the Scientist Expert Agent.

### Google Search Agent

The Google Search Agent provides a rapid, general-purpose information retrieval from the web. It is designed to access a wide array of online sources, including government and clinical websites, news articles, and other publicly available content.

To optimize performance, we desgin a parallel search and summarization architecture. This design addresses key challenges in web-scale data processing, namely: 1) maximizing search throughput; 2) preserving the finite context window of LLMs; and 3) mitigating performance degradation caused by irrelevant or excessively long text.

Our methodology is as follows: An Orchestrator generates multiple search queries, which are executed concurrently via the Google Search API. Each retrieved web page is then routed to a dedicated LLM instance. These instances operate in parallel to summarize their assigned content, guided by a prompt to focus on information relevant to the initial query. This strategy of parallelizing both search and summarization significantly increases processing speed and serves as an effective filter, ensuring that only the most relevant information is retained. Finally, the individual summaries are aggregated and synthesized into a consolidated report that includes robust source citations. Figure 8 illustrates the workflow of the Google Search Agent.

### PubMed Paper Search Agent

The PubMedSearchAgent is a specialized agent to navigate and extract information from the biomedical scientific literature indexed within the PubMed database. Its primary function is to serve as a high-fidelity source for answering complex biomedical questions by retrieving peer-reviewed research articles, clinical studies, and systematic reviews.

The agent’s workflow begins when it receives targeted search queries from the Orchestrator. It utilizes the PubMed API to conduct a comprehensive search, returning a list of relevant publications with their DOIs. Using the list of DOIs, the agent attempts to download the full-text of each article directly from the publisher’s platform. Our current implementation includes major scientific publishers, including PubMed Central (PMC), Elsevier, Wiley, Arxiv, Bioarxiv, and Medrxiv.

We deploy a parallel extraction and summarization strategy to process the dense scientific content efficiently. Each article is assigned to a dedicated LLM instance. These LLMs are prompted to scrutinize the full text, identify, and extract key evidence—such as methodologies, patient cohorts, statistical results, and author conclusions—that is directly relevant to the initial query. This parallel approach ensures that the agent can efficiently process large volumes of literature while isolating the most critical information.

The final output is a synthesized report that includes a summary of the findings from the retrieved articles, along with article titles. Figure 8 illustrates the workflow of the PubMed Paper Search Agent.

### BiomarkerKG Agent

The BioMarkerKG Agent is a specialized biomedical GraphRAG agent that retrieves biomedical subgraphs from a knowledge graph database. This fine-tuned graph language model processes biomedical queries and returns knowledge graph indices, generating subgraphs that contain enriched relationships between biomedical entities. This subgraph, which reveals disease mechanisms, drug-target interactions, or potential biomarkers, is then passed to a large language model (LLM) to synthesize a final, grounded response. Crucially, the BioMarkerKG Agent operates with high priority; its initial subgraph retrieval serves as a grounding framework to inform the search strategies of other agents, such as those for PubMed and web searches.

We construct the BioMarkerKG database using STaRK-Prime (Wu et al. 2024), a large-scale biomedical knowledge graph containing 129,375 entities and 8,100,498 relations across diseases, drugs, and genes/proteins. We convert entities and relations into textual embeddings alongside their descriptions and index the graph database for efficient subgraph retrieval.

We design a RAG QA fine-tuning task using 111,413 biomedical QA pairs from STaRK-Prime’s PrimeKG dataset. For example: Question: “I am looking for a gene or protein that plays a role in ribosomal operations and has an interaction with the protein RPL23. It should be linked to a disorder common with RPL23, and its absence should be a known cause of Diamond-Blackfan anemia. Which gene or protein fits these criteria?” Answer: “2496, 2977, 4933, 59, 717, 1518, 6384, 4789, 2137, 827, 1948, 349, 5054, 7423” (node entity indices).

**Fig. 7.**
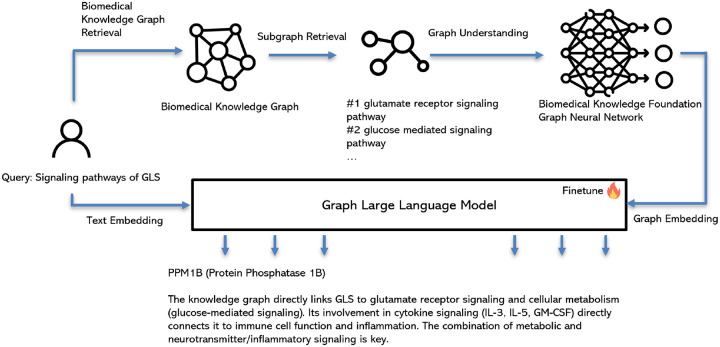
Overview of BioMarkerKG Agent.

**Fig. 8.**
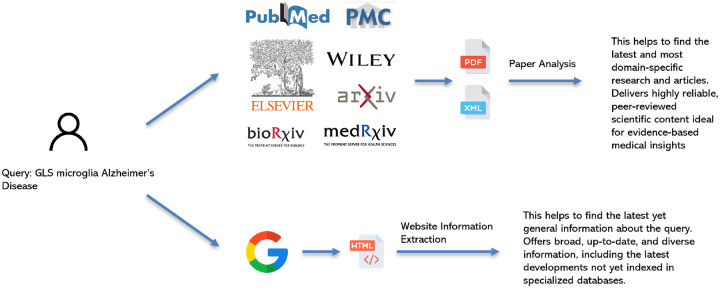
Overview of Pudmed Paper Search Agent and Google Search Agent.

We implement the training using a graph language model (He et al. 2024) specialized for RAG systems, comprising a graph encoder and large language model. The graph encoder employs Graph Neural Network (GNN) architecture, specifically Graph Attention Networks (GAT) (Veličković et al. 2017), paired with Llama 3.1–7b. This architecture effectively encodes graph structure and entity descriptions, enabling the model to understand retrieved subgraph structures and translate them into textual understanding. Fine-tuning on the RAG QA dataset teaches the model to map queries to corresponding entity indices in the graph database. Figure 7 illustrates the workflow of the BioMarkerKG Agent.

## RESULTS

We evaluate the quality and relevance of the text generated by our OmicCellAgent, particularly in comparison to reference texts or ground truths, we employ metrics derived from BERTScore (Zhang et al. 2020). BERTScore leverages contextual embeddings from pre-trained transformer models (specifically BERT) to measure the semantic similarity between candidate (generated) and reference texts.

### We utilize the three standard components of BERTScore:

BERTScore Precision (Bert-P): This metric measures the extent to which tokens in the generated text are semantically similar to tokens in the reference text. High precision indicates that the generated text contains relevant information found in the reference, minimizing hallucination or irrelevant content. BERTScore Recall (Bert-R): This metric assesses how well the generated text covers the semantic content present in the reference text. High recall suggests that the generated text captures most of the important information or key points from the reference. BERTScore F1 (Bert-F1): This is the harmonic mean of BERTScore Precision and Recall, providing a single, balanced measure of overall semantic similarity. It rewards generations that are both relevant (high precision) and comprehensive (high recall) with respect to the reference text.

To evaluate the performance of our system on complex biomedical question answering, we utilized the benchmark dataset and framework from BioASQ Task Synergy 13. This task specifically focuses on biomedical semantic question answering (QA) related to developing health issues. The core challenge requires participating systems to process biomedical questions posed in English. For each question, the system must generate a comprehensive, concise, paragraph-sized summaries in English that synthesize the information into an ideal response. We show our benchmark result in Table 1.

### An example of the question is: Is Hirschsprung disease a mendelian or a multifactorial disorder?

The idea answer is: Coding sequence mutations in RET, GDNF, EDNRB, EDN3, and SOX10 are involved in the development of Hirschsprung disease. The majority of these genes was shown to be related to Mendelian syndromic forms of Hirschsprung’s disease, whereas the non-Mendelian inheritance of sporadic non-syndromic Hirschsprung disease proved to be complex; involvement of multiple loci was demonstrated in a multiplicative model.

## DISCUSSIONS AND CONCLUSIONS

This paper addressed the significant challenge of leveraging AI multi-agent for complex biomedical question answering and knowledge discovery, responding to the need for reliable and up-to-date information synthesis in fields such as precision medicine. We introduced a novel multi-agent system architecture centered around a coordinating Orchestrator agent. The proposed Orchestrator-driven multi-agent system offers a valuable architectural approach for building more capable, transparent, and trustworthy AI systems poised to accelerate progress in biomedical research and precision medicine.

In addition to the existing scRNAseq data analysis, one of the fundamental challenges is to identify the key signaling targets and networks from a large-set of differentially expressed genes. In previous studies(Zhang et al. ; Dong et al. 2023; Feng et al. 2024b; Feng et al. 2024a; Zhang et al. 2024b; Zhang et al. 2024a; Zhang et al. 2024c; Song et al. 2025), we have developed multiple graph models that systematically integrate omic data with signaling pathways or protein-protein interaction signaling networks. In the future work, we will develop incorporate the graph AI or graph foundation models into omniCellAgent for identifying the key signaling targets and core pathways, as well as drugs and drug combinations that can perturb the uncovered signaling pathways.

## Figures and Tables

**Fig. 2.**
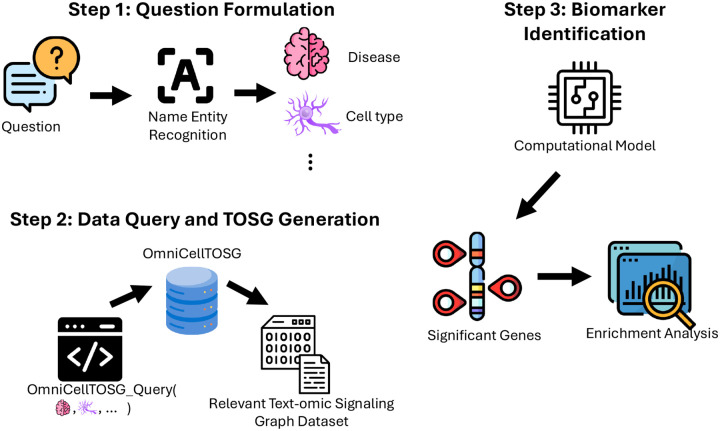
Overview of the Omic Data Agent Workflow.

**Figure:**
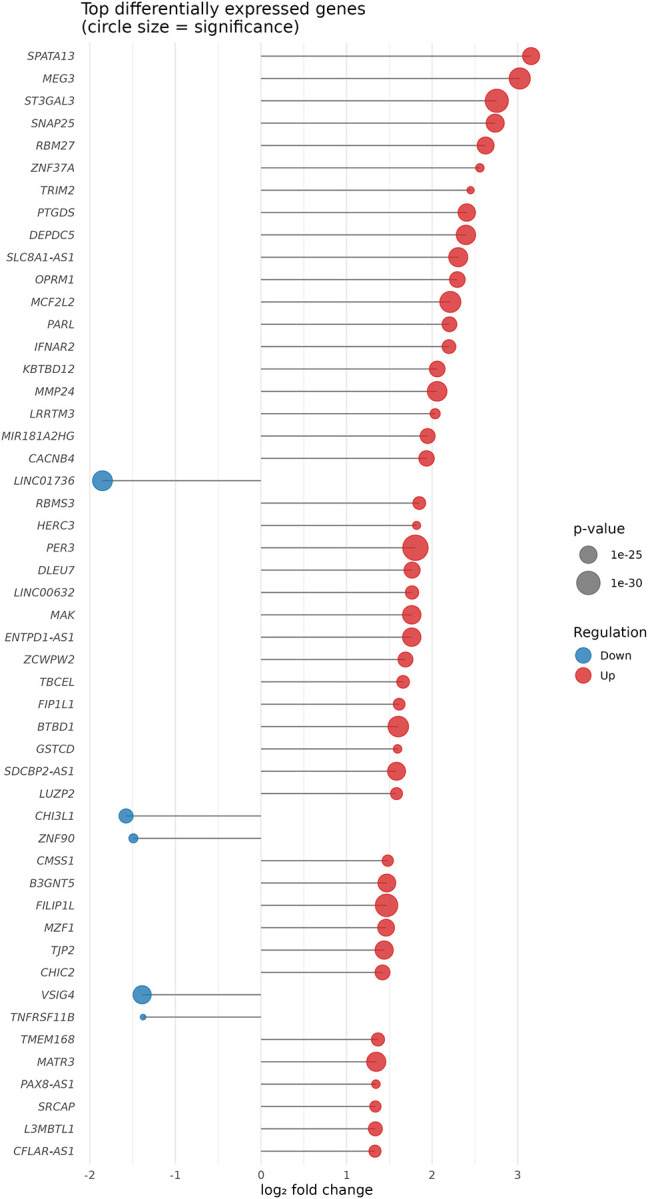


**TABLE 1.** Performance Metrics for BERTScore

	Bert-F1	Bert-P	Bert-R
Average	0.887119	0.877531	0.897939
